# Therapeutic drug monitoring: linezolid too?

**DOI:** 10.1186/s13054-014-0525-x

**Published:** 2014-09-15

**Authors:** Guy A Richards, Adrian J Brink

**Affiliations:** Division of Critical Care, University of the Witwatersrand and Charlotte Maxeke Johannesburg Academic Hospital, Faculty of Health Sciences, University of the Witwatersrand, York Road, Parktown, 2193, Johannesburg, South Africa; Department of Clinical Microbiology, Ampath National Laboratory Services, Suite 9C, Milpark Hospital, 9 Guild Road, Parktown West, 2193, Johannesburg, South Africa

## Abstract

Numerous factors interfere with the ability to achieve optimal pharmacokinetic and pharmacodynamic targets and this has been associated with greater mortality and lower cure rates. The recent study by Zoller and colleagues examining linezolid levels in critically ill patients emphasises this point. Their study is unique in the description of the intra-patient and inter-patient variability that occurs and in the degree to which therapy is inadequate; 63% of patients had insufficient levels and only 17% maintained optimal trough values (between 2 and 10 mg/l) throughout the 4 study days. Precisely why this result occurred is uncertain because albumin levels, free linezolid pharmacokinetics and the presence of augmented renal clearance were not recorded in the current study. The extent of this variability makes the case for therapeutic drug monitoring since an area under the inhibitory curve greater than 80 to 120 and the time above the minimum inhibitory concentration over the entire dosing interval strongly correlate with linezolid treatment efficacy. Accordingly, therapeutic drug monitoring where available or, if not available, alternative approaches to drug delivery such as continuous infusion or a dose increase – but particularly the former – may be the answer.

Antibiotic dosing of critically ill patients is notoriously difficult because multiple factors influence achievement of pharmacokinetic and pharmacodynamic targets. Linezolid (LZD) is no exception to this and strategies need to be developed to ensure therapeutic adequacy.

The study by Zoller and colleagues in a previous issue of *Critical Care* [1]*,* who examined LZD levels in 30 critically ill patients, highlights this issue. Whilst therapeutic drug monitoring (TDM) has traditionally been used to minimise toxic effects, the study results support emerging data that TDM should also be used to optimise dosing in critically ill patients.

Numerous factors such as obesity, volume of distribution, albumin levels, fluid losses and whether the drug is hydrophobic or hydrophilic interfere with the ability to achieve optimal pharmacokinetic and pharmacodynamic targets and subsequent bacteriological and clinical success. The free fraction is responsible for efficacy and toxicity but is also available for clearance. In addition, the phenomenon of augmented renal clearance has been associated with greater mortality and lower cure rates, most marked with creatinine clearance ≥150 ml/minute [2].

Whereas previous studies have investigated the pharmacokinetic/pharmacodynamic parameters of LZD and have described the difficulties associated with predicting serum and tissue levels, the study by Zoller and colleagues is unique primarily in the number of evaluations that were performed and in the description of intra-patient and inter-patient variability. The area under the concentration–time curve for a drug is the basis on which the area under the inhibitory curve (AUIC; the best pharmacodynamic marker of efficacy for LZD) is calculated, and the trough concentration is the concentration from which the time above the minimum inhibitory concentration (T > MIC) is calculated using the breakpoints of common Gram-positive pathogens. Both of these parameters showed marked variability in this study and, using the AUIC as a predictor of adequacy of therapy, 63% of patients had insufficient levels. Similarly, only 17% of patients maintained optimal trough concentration values (between 2 and 10 mg/l) throughout the 4 study days. Precisely why this result occurred is uncertain.

In a LZD pharmacokinetics study, Lovering and colleagues demonstrated that although the volume of distribution and renal clearance were similar between healthy volunteers and severe burn patients, nonrenal clearance was substantially increased in the burn patients – resulting in a lower area under the concentration–time curve, possibly due to loss of drug in burn exudate and thereafter due to changes in basal metabolism [3]. Although this situation was not the case here, nonrenal clearance might have played a role. LZD is moderately protein bound (±30%), so the impact of hypoalbuminaemia on target attainment would be expected to be less than that of highly protein-bound drugs. Wong and colleagues, however, recently demonstrated variabilities in both unbound and total concentrations that were significant for all β-lactam antibiotics whether or not they were highly protein bound [4]. In addition, in a recent multicentre study 16% of 248 critically ill patients had T > MIC for free β-lactam levels <50% and a positive clinical outcome was associated with increasing the T > MIC to 50% (odds ratio, 1.02) and to 100% (odds ratio, 1.56) (*P* < 0.03) [5]. In the current study, albumin levels, free LZD pharmacokinetics and augmented renal clearance were not recorded.

The considerable and unpredictable inter-patient and intra-patient variability recorded by Zoller and colleagues certainly makes the case for TDM, as AUIC >80 to 120 and T > MIC over the entire dosing interval strongly correlate with LZD treatment efficacy [6]; unfortunately, TDM is not yet generally available. Until it does become available, we need to develop more generally applicable alternative approaches to drug delivery. One of these approaches might be continuous infusion, which reliably increases levels above the breakpoint of 2 mg/l and avoids wide swings in peak and trough levels [7]. In support of this hypothesis, Boselli and colleagues demonstrated that a loading dose followed by continuous infusion led to concentrations twice that of a LZD minimum inhibitory concentration of 4 mg/l in serum and epithelial lining fluid for 100% of the time in critically ill patients with ventilator-associated pneumonia [8]. Clearly, a lot more work is necessary to confirm that this is the situation in critically ill patients despite the alterations in physiological parameters that occur. Alternatively, could increasing the dose be the answer? Infusion of 1,800 mg/day LZD should reliably increase levels into the therapeutic range but would also increase toxicity [9]. In the current study, toxic levels occurred in 7% of patients utilising the recommended dose, with no factors predictive of risk other than a trend for higher levels with an elevated creatinine level.

In conclusion, optimising the dose and method of administration of LZD is essential. Whereas further studies of both approaches described above are necessary before firm recommendations can be made, it seems reasonable to utilise TDM where available, which in future might warrant utilising free antibiotic levels – and, where TDM is not available, continuous infusion might significantly improve AUIC.

## Abbreviations

AUIC: Area under the inhibitory curve
LZD: Linezolid
TDM: Therapeutic drug monitoring
T > MIC: Time above the minimum inhibitory concentration
